# Computational method using heterogeneous graph convolutional network model combined with reinforcement layer for MiRNA–disease association prediction

**DOI:** 10.1186/s12859-022-04843-3

**Published:** 2022-07-25

**Authors:** Dan Huang, JiYong An, Lei Zhang, BaiLong Liu

**Affiliations:** School of Computer Science and Technology, China University of Mining and Technology, Xuzhou, 21116 Jiangsu China

**Keywords:** miRNA and disease interactions, Graph convolutional network

## Abstract

**Background:**

A large number of evidences from biological experiments have confirmed that miRNAs play an important role in the progression and development of various human complex diseases. However, the traditional experiment methods are expensive and time-consuming. Therefore, it is a challenging task that how to develop more accurate and efficient methods for predicting potential associations between miRNA and disease.

**Results:**

In the study, we developed a computational model that combined heterogeneous graph convolutional network with enhanced layer for miRNA–disease association prediction (HGCNELMDA). The major improvement of our method lies in through restarting the random walk optimized the original features of nodes and adding a reinforcement layer to the hidden layer of graph convolutional network retained similar information between nodes in the feature space. In addition, the proposed approach recalculated the influence of neighborhood nodes on target nodes by introducing the attention mechanism. The reliable performance of the HGCNELMDA was certified by the AUC of 93.47% in global leave-one-out cross-validation (LOOCV), and the average AUCs of 93.01% in fivefold cross-validation. Meanwhile, we compared the HGCNELMDA with the state‑of‑the‑art methods. Comparative results indicated that o the HGCNELMDA is very promising and may provide a cost‑effective alternative for miRNA–disease association prediction. Moreover, we applied HGCNELMDA to 3 different case studies to predict potential miRNAs related to lung cancer, prostate cancer, and pancreatic cancer. Results showed that 48, 50, and 50 of the top 50 predicted miRNAs were supported by experimental association evidence. Therefore, the HGCNELMDA is a reliable method for predicting disease-related miRNAs.

**Conclusions:**

The results of the HGCNELMDA method in the LOOCV (leave-one-out cross validation, LOOCV) and 5-cross validations were 93.47% and 93.01%, respectively. Compared with other typical methods, the performance of HGCNELMDA is higher. Three cases of lung cancer, prostate cancer, and pancreatic cancer were studied. Among the predicted top 50 candidate miRNAs, 48, 50, and 50 were verified in the biological database HDMMV2.0. Therefore; this further confirms the feasibility and effectiveness of our method. Therefore, this further confirms the feasibility and effectiveness of our method. To facilitate extensive studies for future disease-related miRNAs research, we developed a freely available web server called HGCNELMDA is available at http://124.221.62.44:8080/HGCNELMDA.jsp.

## Background

As a kind of non-coding RNA with regulatory properties and highly conserved in the evolutionary process, miRNA is approximately 20–24 nucleotides in length. Researchers that have been studying miRNA [1] have found that it plays a vital role in biological processes such as cell growth, proliferation, metabolism, differentiation and apoptosis. Moreover, the abnormal expression of miRNA has also been proved to be closely related to some diseases, such as chronic lymphocytic leukemia, tumor, gastric cancer, cardiomyopathy, etc. Therefore, identifying the correlation between miRNA and diseases has become a critical step in biological research recently [2]. However, the traditional biological experiments take up a long time, cost much, and have some blindness, all of which would stall the research process. Therefore, many researchers are devoted to designing computational methods to discover the interaction between unidentified miRNAs and diseases to make up for the shortcomings of traditional experimental approaches [3].

Currently, researchers have established a series of effective calculation models for miRNA–disease association prediction, which can be roughly divided into two categories according to the methods used: similarity measurement-based and machine learning-based. For similarity measurement [4], the miRNA–disease association is predicted by measuring the degree of similarity between nodes using different statistical methods. The machine-learning approach trains other models by learning features and then predicting miRNA–disease associations based on the trained models. The above two methods have different theoretical bases and innovations, and thus making outstanding contributions to future research. For example, Jiang et al. [] determined the functional correlation of two miRNAs by calculating the number of familiar neighbors and the shortest path length of two miRNAs and constructing two miRNAs' functional correlation information. For the first time, Jiang et al. combined disease phenotype information with miRNA function information to predict miRNA–disease association [5], contributing significantly to the future research. Subsequently, for each predicted disease, they designed a hypergeometric distribution-based scoring system [6] to score the diseases and all of the miRNAs associated with them. However, this method comes with some limitations too. Because only the direct neighbors of the miRNA were considered as the criterion for miRNA functional similarity score, the prediction effect was limited. To increase the accuracy of miRNA–disease association prediction, Xuan et al. [7] proposed the weighted k-nearest neighbor method (HDMP). Chen et al. developed the computational framework of RWRMDA that performs random walk on the miRNA network to predict novel disease-related miRNAs. They first put the initial probability values on the pre-constructed miRNA functional similarity network (MFSN) to conduct random walk algorithm. In summary, this model integrated miRNA functional similarity and known miRNA–disease associations to infer novel disease-related miRNAs. They suggested that members of the same miRNA family may be involved in diseases with related phenotypes. According to the association state of the nearest neighbor [8], members of the miRNA family and miRNA cluster can obtain more weight, which improves the prediction performance of the model to some extent. However, it is difficult to manually select the optimal parameter K that classifies the number of members in each miRNA family and miRNA cluster [9], and this method cannot predict new diseases that do not have known miRNA associations. Pasquier et al. [10] formed a matrix with higher dimensions based on miRNA–disease association, miRNA target association, miRNA word association, miRNA family association and miRNA neighbor association state data. Using the singular value matrix decomposition method to decompose the matrix, Pasquier et al. successfully obtained miRNA vectors and disease vectors [11]. They took the cosine distance between the miRNA node vector and the disease node vector as the degree of association between the nodes. However, due to the false-positive rate and false-negative rate between miRNA and target, the model's prediction performance is affected to a certain extent. In WBSMDA [12], authors integrated comprehensive similarity score between the miRNA and disease based on Gaussian interaction profile kernel. WBSMDA could be applicable to the new miRNAs without disease association and to diseases without miRNA association, thereby overcoming the previous limitation of the prediction model.

In addition to similarity-based approaches, machine learning algorithms aiming at exploring potential miRNA disease interactions are also an essential academic approach in this field. Unlike the method of directly calculating the similarity between nodes in the network based on similarity itself, the machine learning approach [13] is devoted to extracting inherent features and designing practical classification algorithms to find miRNA and disease associations. As an early method based on machine learning, Jiang et al. [14] first extracted feature vectors from disease similarity and miRNA function similarity. Then, they randomly selected 270 samples from unknown miRNA disease pairs as negative data, as missing negative instances in the actual data set [15]. Finally, they chose the SVM (support vector machine) as the classifier [16]. However, this artificial method randomly selected negative samples, impacting on the model's accuracy. A different approach conducted by Chen et al. [17] constructed a semi-supervised classifier with regularized least squares. Although the model does not require negative samples, and the possibility of unknown associations is confirmed, this method also has some limitations: the predicted results of fusion miRNA and disease are strongly dependent on parameters [18], and thus it is difficult to choose the optimal parameters. Chen et al. [19] proposed the DRMDA method to use stacked autoencoders for feature extraction to obtain low-dimensional and high-resolution feature vectors and then used SVM to score candidate miRNAs. This method eliminated a lot of noise in similar unprocessed data and achieved good performance results. Graph neural network has attracted extensive attention from researchers due to its high precision. Li et al. Presented a model of MCMDA that exploited known miRNA–disease associations to build binary adjacency matrix, and imple mented a singular value thresholding (SVT) algorithm to extract miRNA–disease associations [20]. Pasquier et al. [21] made the assumption that information attached to miRNAs and diseases can be revealed by distributional semantics. The approach represented distributional information on miRNAs and diseases in a high-dimensional vector space and defined associations between miRNAs and diseases in terms of vector similarity. Chen et al. proposed a prediction model of ensemble of decision tree-based miRNA–disease association (EDTMDA). This model adopted dimensionality reduction algorithm for principal component analysis (PCA) to apply ensemble learning to predict disease-related miRNAs [22]. Ha et al. [23] proposed it focuses on the problem of inferring miRNA and disease associations by exploiting distance metric learning on miRNA–disease bipartite graph, which is constructed based on the known miRNA–disease associations.

Also, biological information networks such as disease and miRNA have complex topological structures, so it is suitable for graphical modelling [24]. For graph data, graph convolutional networks (GCN) have better performance than inhomogeneous networks (such as classification). Therefore, researchers have been trying to apply GCN in heterogeneous networks to predict the association between miRNA and disease [25]. For example, Li et al. [26] extracted node features from the protein–protein interaction network and put them into the graph convolutional network following the Node2VEC algorithm. Finally, each node was embedded in the graph convolutional layer, and the miRNA–disease association was obtained by multiplying the miRNA–gene adjacency matrix by the disease-gene adjacency matrix [27]. This method provides a new perspective for the field of miRNA–disease association prediction. Then, Li et al. [28] proposed the FCGCNMDA method based on a fully connected graph. They extracted the aggregation of node features by using a two-layer graph convolution layer in miRNA functional similarity network and disease semantic similarity network to make end-to-end prediction [29]. However, the GCN model considers all neighbors equally, and the similarity information of nodes cannot be retained when learning node embedding. Li et al. presented [30] a model of MCMDA that exploited known miRNA–disease associations to build binary adjacency matrix, and implemented a singular value thresholding (SVT) algorithm to extract miRNA–disease associations. However, choosing the best parameters of the algorithm restricted to any further improvement in prediction accuracy. Ha et al. [31] proposed focuses on the problem of inferring miRNA and disease associations by exploiting distance metric learning on miRNA–disease bipartite graph, which is constructed based on the known miRNA–disease associations.

Although the existing methods have good performances in predicting miRNA–disease associations, we can still improve some aspects of them. On the one hand, some methods [32] produce inevitable data noise during feature extraction, affecting the prediction effect. On the other hand, some graph [33] convolution methods fail to retain the similarity information of nodes so that similar nodes have similar feature representations in the feature space to enhance the spatial node features of the topology graph [34]. This paper is based on strengthening layer figure convolution heterogeneous network model HGCNELMDA (heterogeneous graph convolutional network model with enhanced layer to predict miRNA–disease associations) to extract node features from the level of the graph. To reduce the data noise of the similarity matrix calculation, the random reboot walk is used to get the original features of nodes from the similarity matrix. Graph convolution aggregates node information according to edge information and represents new node features. Before the figure of convolution model, GCN (graph convolutional network) will consider all equal neighbors, and thus being unable to retain when learning node embedded nodes similarity information. The enhancement layer added in the GCN hidden layer is used to strengthen the similar representation of similar nodes (miRNAs or diseases) in the feature space and enhance the eigenvector aggregation of similar nodes to retain similar information between nodes. First, we constructed an miRNA–disease heterogeneous network based on the proven miRNA–disease association, disease semantic similarity and miRNA functional similarity. Second, to reduce the data noise of extracting the original feature vectors of miRNA and disease nodes and better capture the structural relationship between different types of nodes in heterogeneous graphs, the method based on restart random walk is used for extracting node features from similarities. Third, the miRNA–disease heterogeneous graph and the miRNA–disease feature matrix are gathered through graph convolution to gather the information of neighbor nodes on the layer, and an attention-based reinforcement layer is added to the hidden layer. In the miRNA–disease heterogeneous graph, to strengthen similar nodes (miRNA or disease) for similar representations in the feature space, a reinforcement layer is added to the GCN hidden layer, enhancing the feature vectors of similar aggregate retain similar information between nodes. The attention mechanism is introduced in the reinforcement layer, and more critical topological neighborhood nodes are merged, and miRNA and disease node features are extracted from the spatial topological structure of heterogeneous graphs to predict associations. The results of the HGCNELMDA method in LOOCV (leave-one-out cross-validation) and fivefold cross-validations were 93.47% and 93.01%, respectively. Compared with other typical methods, the HGGCNMA has a better performance. Four cases of lung cancer, prostate cancer and pancreatic cancer were used for research. Among the predicted top 50 candidate miRNAs, 48, 50, and 50 were verified in the biological database HDMI V2.0. Therefore, the result further confirms the feasibility and effectiveness of our method.

## Results

First, we present the experimental methods and evaluation indexes. The performance of the HGCNELMDA approach is then compared with the following four existing approaches. Finally, we used the HGCNELMDA method to determine the accuracy of the predictive association based on three cases of prostate tumor, lung tumor and pancreatic tumor.

### Experimental approaches and evaluation criteria

We collected 5430 known miRNA–disease associations from HMDD V2.0 as the experimental data set. Based on experimentally verified associations between miRNAs and diseases, we implemented global LOOCV and fivefold CV to evaluate the predictive accuracy of HGCNELMDA. In LOOCV evaluation, every confirmed association was regarded as a test sample in turn, while the rest associations were treated as training samples. In general, two types of LOOCV exists (global LOOCV, local LOOCV). Global LOOCV considers all the diseases at the same time while local LOOCV only take account of the miRNAs for a given disease of interest. Candidate samples included all of the miRNA–disease pairs that experimental studies had not verified. After executing HGCNELMDA, every miRNA–disease pair will obtain an association score. A higher score means a higher likelihood for a link to exist between a pair. In global LOOCV, we compared the score of the test sample with the scores of all the candidate samples. Furthermore, we drew receiver operating characteristics (ROC) curve by plotting the actual positive rate (TPR, sensitivity) against the false positive rate (FPR, 1-specificity) at different thresholds. Sensitivity denotes the percentage of miRNA–disease test samples with ranks exceeded the given point, while specificity represents the percentage of negative miRNA–disease associations with ranks lower than the threshold. AUC was further calculated to demonstrate the prediction ability of HGCNELMDA. The model has perfect prediction performance when AUC reaches exactly 1. If AUC equals 0.5, it suggests that the model only has random prediction performance.

Moreover, we exploited fivefold CV to examine the predictive accuracy further. Fivefold cross-validation was also implemented to further estimate the prediction accuracy of the HGCNELMDA model by randomly dividing the known associations equally into five groups and treating each one of them as test samples in turn by removing the associations of the current test samples simultaneously. Afterwards, every test sample would be scored and compared with the candidate miRNA–disease pairs to obtaining the rankings. We repeated this procedure 50 times to get a more accurate average AUC value.

### Compare with other methods

**Fig. 1:**
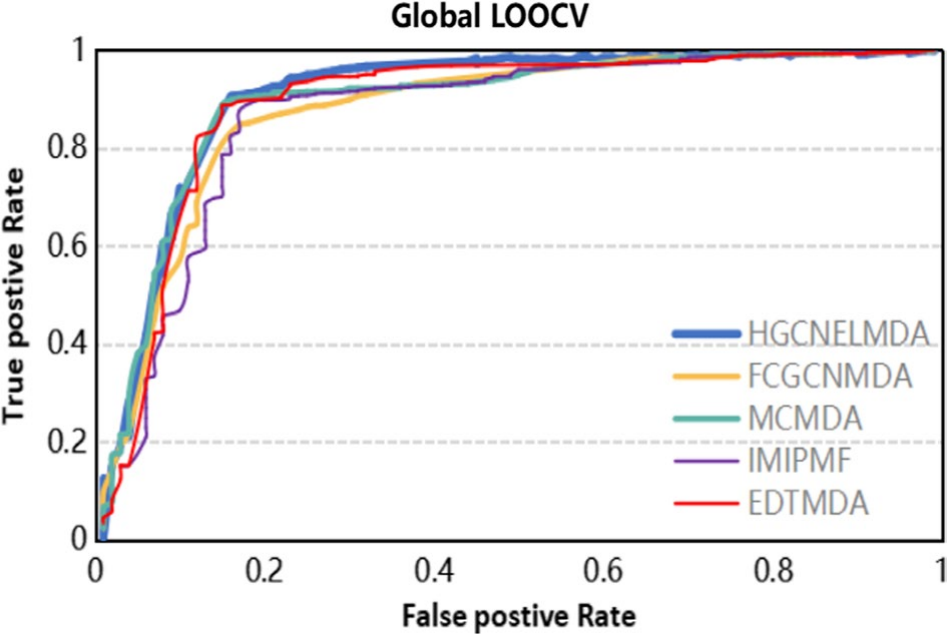
Comparison of HGCNELMDA and other models for Global LOOCV

**Fig. 2:**
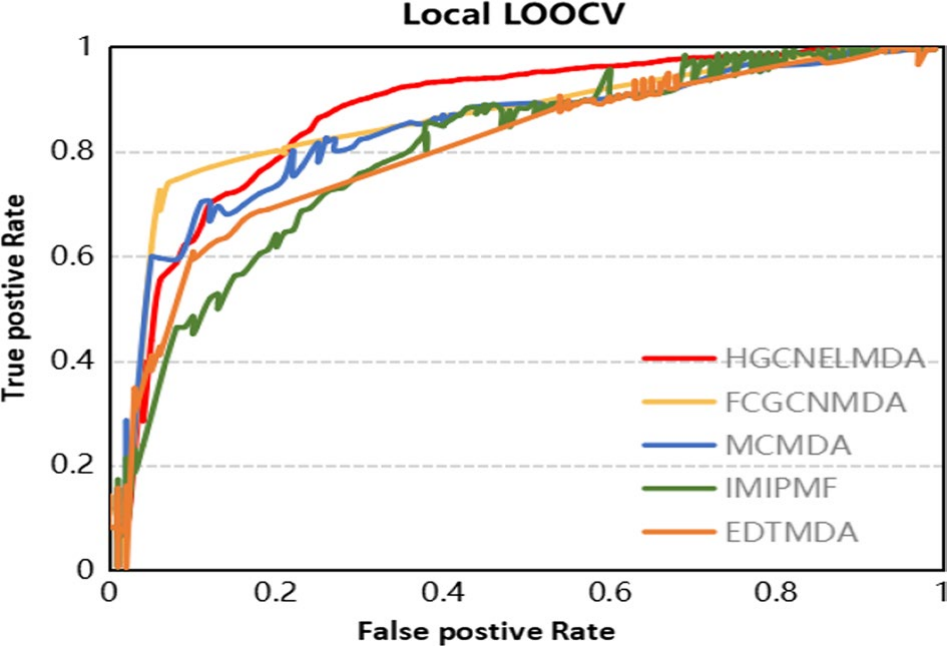
Comparison of HGCNELMDA and other models for Local LOOCV

In order to verify the accuracy of our method, the HGCNELMDA method was compared with the following four existing methods, namely FCGCNMDA [35], CNMDA [36], EDTMDA [37], MCMDA [20], IMIPMF [38] and RKNNMDA [39], for fivefold cross-validation. As shown in Table 1, the AUC of FCGCNMDA, CNMDA, EDTMDA, MCMDA, IMIPMF and RKNNMDA were 92.85%, 85.33%, 91.92%, 86.47%, 89.32% and 82.21%, respectively. Among them, the AUC of HGCNELMDA was the highest under fivefold cross-validation, with a value of 93.01% , the AUPR value of HGCNELMDA was 85.37% and the ACC value of HGCNELMDA was 84.36%. Therefore, HGCNELMDA was proved to be reliable in miRNA–disease association. As for global LOOCV, MLMD achieved a reliable AUC value of 0.8786, which was also superior to that in FCGCNMDA (0.8964), MCMDA (0.8629), IMIPMF (0.8857), and EDTMDA (0.8878), as shown in Fig. 1. As shown in Fig. 2, our model showed superior performance (AUC value 0.8634) compared to FCGCNMDA (0.8596), MCMDA (0.8561), IMIPMF (0.8547), and EDTMDA (0.8512) in the framework of local LOOCV.

**Table 1:** Comparison of HGCNELMDA and other models for fivefold cross-validation

Control group	AUC (%)	AUPR (%)	ACC (%)
**HGCNELMDA**	**93.01**	**85.37**	**84.36**
FCGCNMDA	92.85	83.69	82.51
CNMDA	85.33	76.31	75.17
EDTMDA	91.92	83.92	83.25
RKNNMDA	82.21	73.83	72.92
MCMDA	86.47	75.62	73.72
IMIPMF	89.32	80.56	81.36

### Comparison of results with or without reinforcement layer

**Fig. 3:**
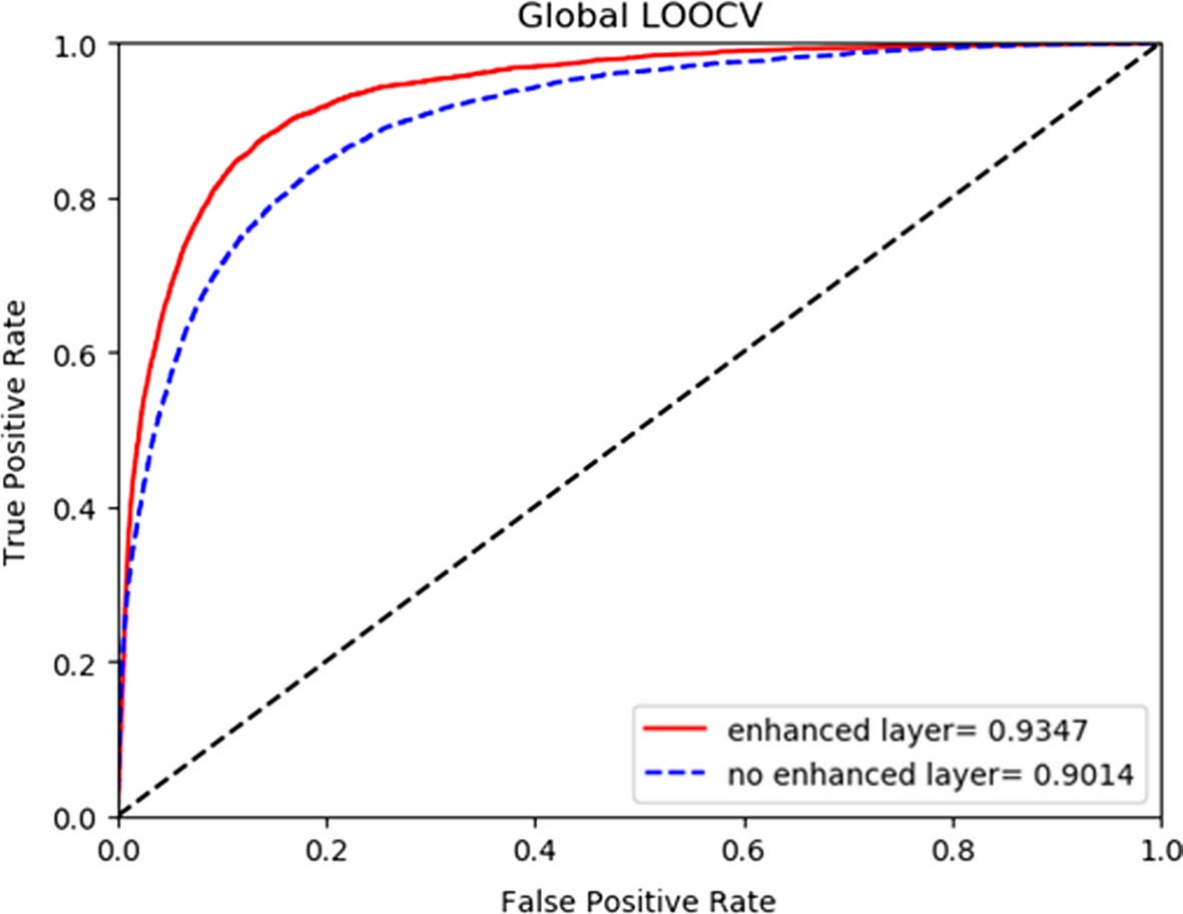
Comparison of left cross-validation with or without reinforcement layer

**Fig. 4:**
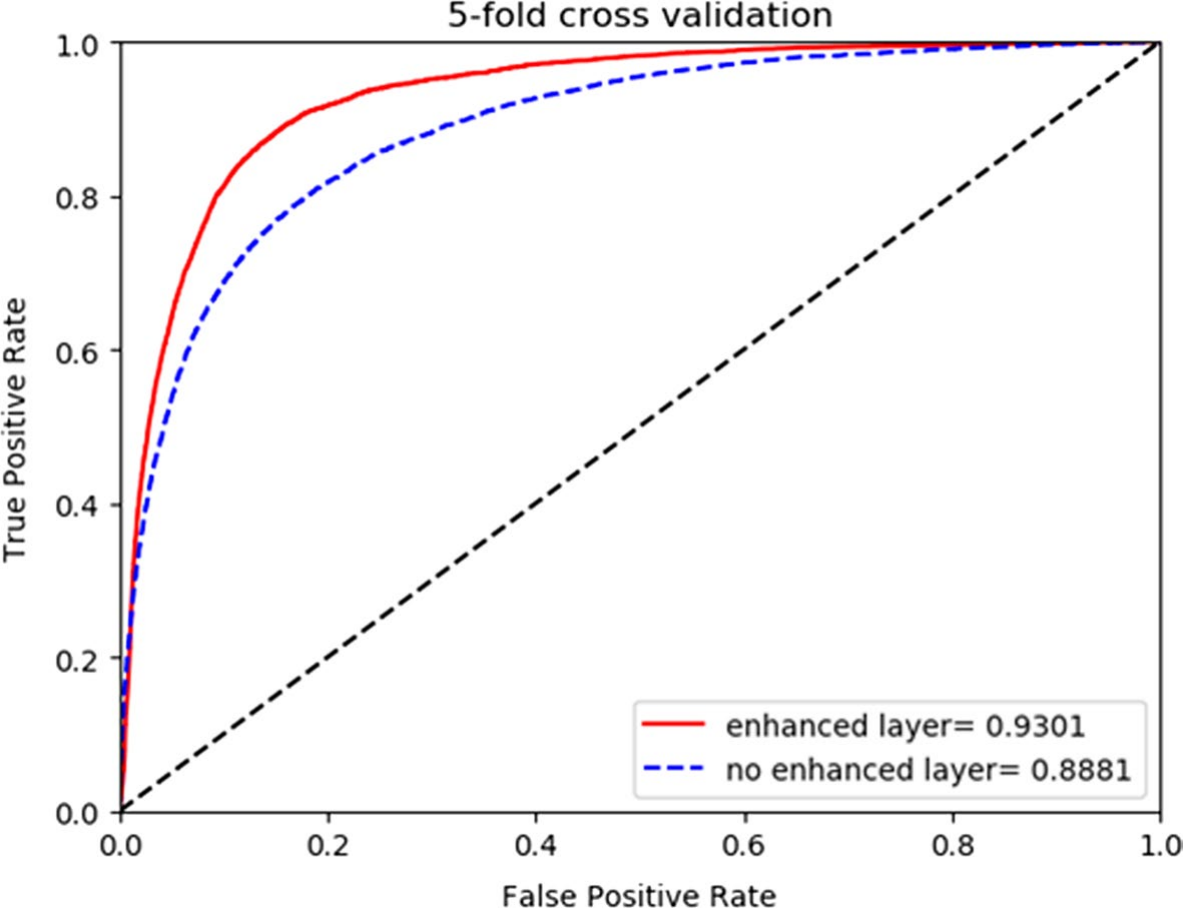
Comparison of fivefold cross validation with or without reinforcement layer

Figures 3 and 4 respectively show the influence of HGCNELMDA on the model performance with or without reinforcement layer under onefold cross-validation and fivefold cross-validation. In the experiment, the reinforcing layer is removed and replaced by the common hidden layer of GCN. The results showed that the AUC value with the reinforcement layer was higher than that without the hidden layer, because the similar miRNA (or disease) nodes in the reinforcement layer were similar in the feature space, and the attention mechanism was used to focus on the aggregation of similar important neighbor nodes in the reinforcement layer, and the similar information of nodes was retained.

### Comparison of results with or without random walk with restart

**Fig. 5:**
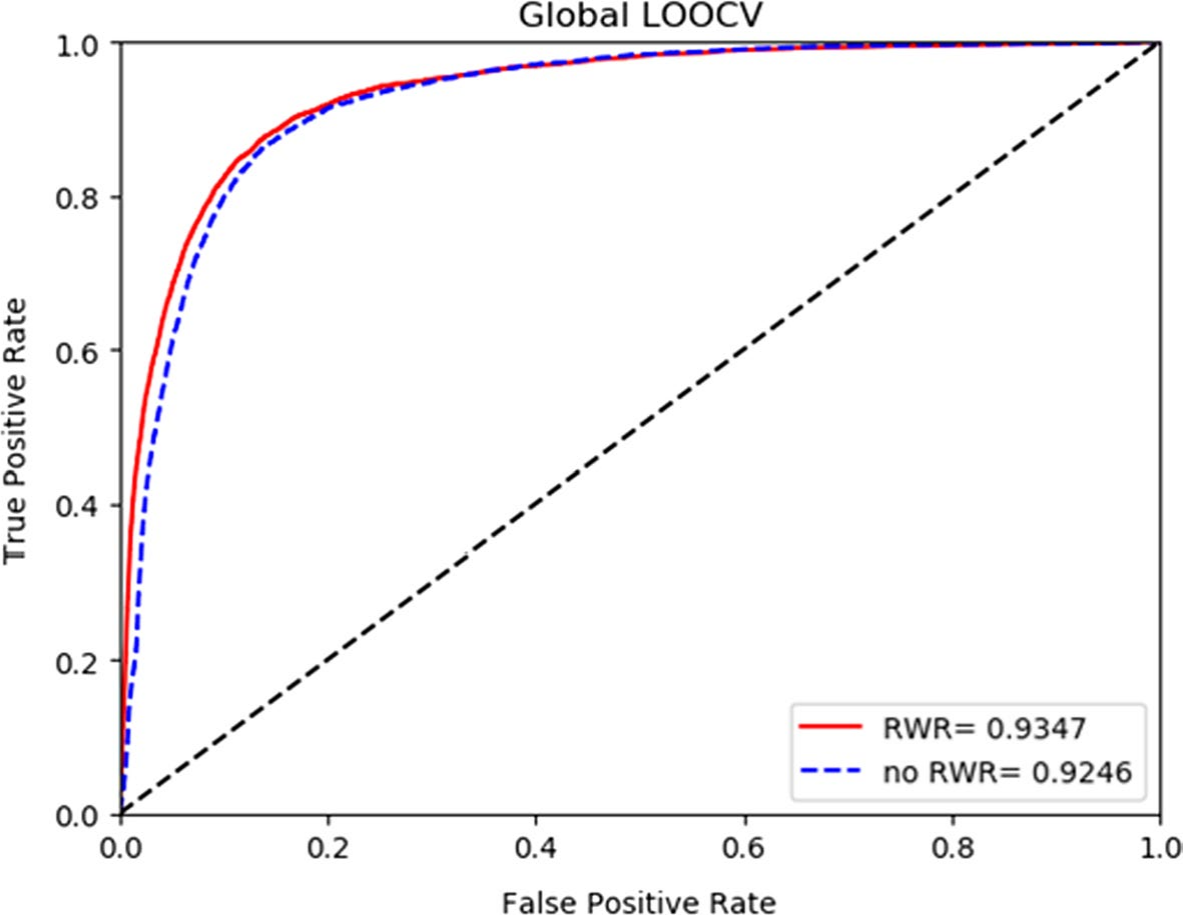
Comparison of residual cross-validation with or without RWR

**Fig. 6:**
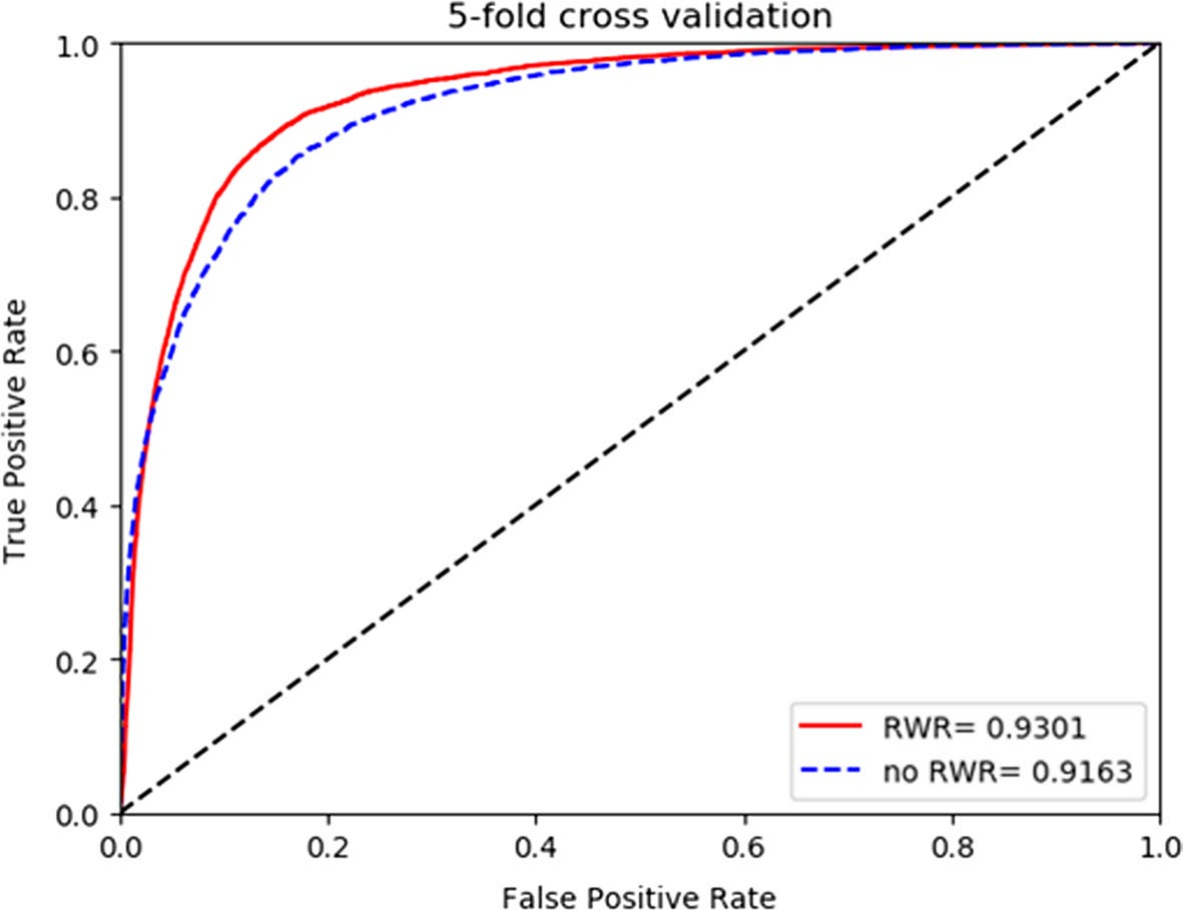
Comparison of fivefold cross validation with or without RWR

Figures 5 and 6 respectively show the influence of HGCNELMDA on the results by using RWR to extract node features under onefold and fivefold cross validation. No experiments using RWR were used directly $SM$ and $SD$ a row or a column of is used as the eigenmatrix of nodes. As shown in the figure, it is better to use RWR as the initial feature of the node, because RWR can select adjacent nodes to travel or return to the initial node, thus reducing the influence of data noise in node feature extraction.

### Comparison of parameter sensitivities

**Fig. 7:**
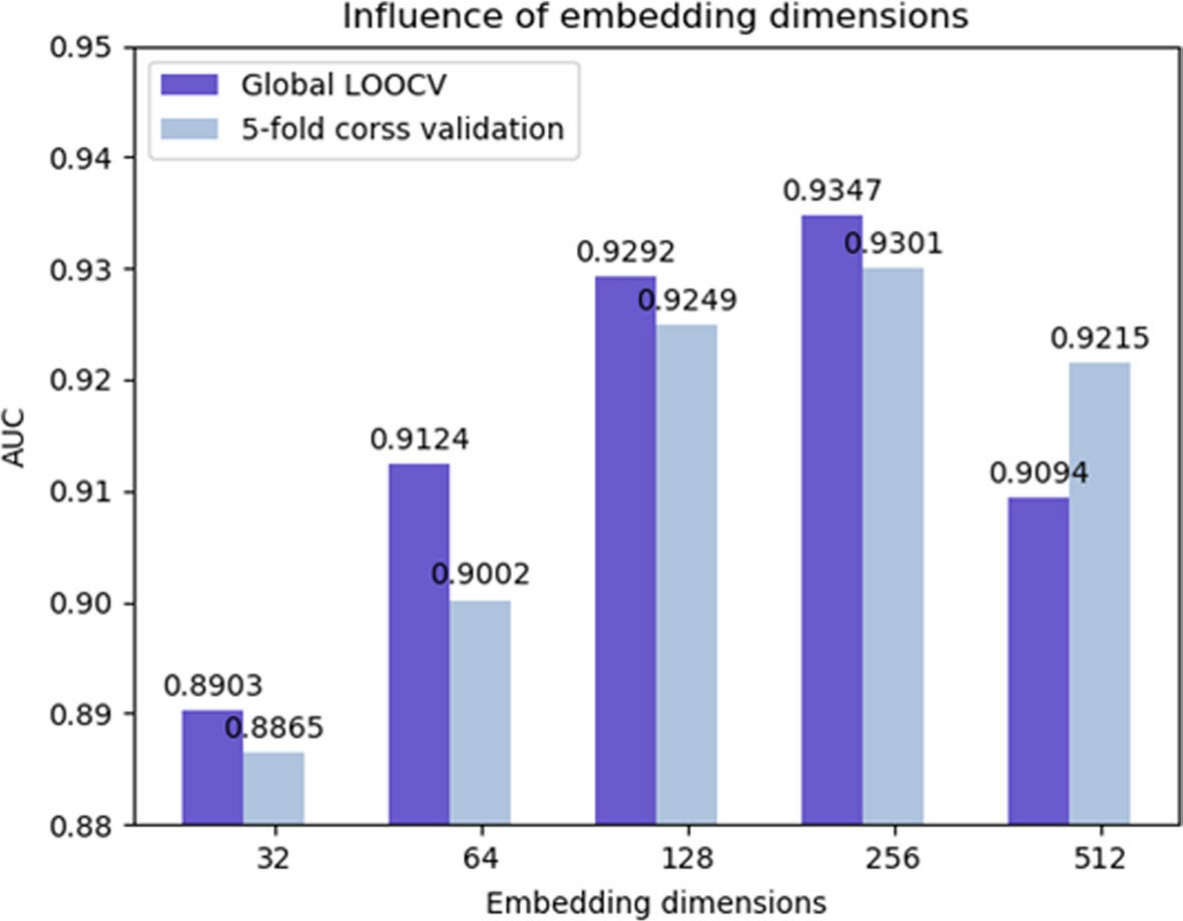
Comparison of different embedded dimensions

Layer node embedding dimension is the node embedding parameter in GCN hidden layer $h$, Different parameter values will affect the experimental results. As shown in Fig. 7, define $h$ as [32, 64, 128, 256, 512], Compared with the AUC results, The validation methods of one-left cross-validation and fivefold cross-validation show that the AUC value presents an upward trend with the increase of node embedding dimension $h$. The performance of the HGCNELMDA approach is highest when the embedding dimension $h$ is defined as 256.

### Cases studies

The HGCNELMDA method was used to determine the accuracy of the predictive association based on three cases of prostate cancer, lung cancer and pancreatic cancer. We compared the predicted candidate miRNAs with DBDEMC and Phenomir, two public databases, to verify their accuracy.

In the first case study, the selected prostate tumors are used to test whether our approach is suitable for novel diseases with unsupported miRNAs or not. This case selected prostate tumors because this is the most common cancer happening on males worldwide. In 2018, more than 100,000 males died of prostate cancer in Europe alone [40]. This case study first set all miRNA–disease associations related to prostate neoplasms from HMDD 2.0 to zero. Then, M2GMDA was performed to identify the associated miRNAs for prostate neoplasms. Table 2 lists the top 50 candidate miRNAs for HGCNELMDA prediction associated with prostate tumors. The first 50 miRNAs were verified by DBDEMC and Phenomir databases. The results show that the above two databases could verify the first 50 miRNAs.

**Table 2:** Top 50 miRNAs associated with prostate tumors

miRNA	dbDEMC	PhenomiR	miRNA	dbDEMC	PhenomiR
hsa-mir-10a	Confirmed	Confirmed	hsa-mir-297	Confirmed	Confirmed
hsa-mir-96b	Confirmed	Confirmed	hsa-mir-23a	Confirmed	Confirmed
hsa-mir-186	Confirmed	Confirmed	hsa-mir-27a	Confirmed	Confirmed
hsa-mir-194	Confirmed	Confirmed	hsa-mir-33b	Confirmed	Confirmed
hsa-mir-15a	Confirmed	Confirmed	hsa-mir-19a	Confirmed	Confirmed
hsa-mir-26b	Confirmed	Confirmed	hsa-mir-1	Confirmed	Confirmed
hsa-let-7d	Confirmed	Confirmed	hsa-mir-27b	Confirmed	Confirmed
hsa-mir-20a	Confirmed	Confirmed	hsa-mir-218	Confirmed	Confirmed
hsa-mir-301a	Confirmed	Confirmed	hsa-let-7e	Confirmed	Confirmed
hsa-mir-363	Confirmed	Not confirmed	hsa-mir-373	Confirmed	Confirmed
hsa-mir-23b	Confirmed	Confirmed	hsa-mir-16	Confirmed	Confirmed
hsa-mir-92	Confirmed	Confirmed	hsa-mir-197	Confirmed	Confirmed
hsa-mir-302d	Confirmed	Confirmed	hsa-mir-181b	Confirmed	Confirmed
hsa-mir-195	Confirmed	Confirmed	hsa-mir-23b	Confirmed	Confirmed
hsa-mir-130b	Confirmed	Confirmed	hsa-mir-101	Confirmed	Confirmed
hsa-let-7i	Confirmed	Confirmed	hsa-mir-26a	Confirmed	Confirmed
hsa-let-7c	Confirmed	Confirmed	hsa-mir-17	Confirmed	Confirmed
hsa-mir-92a	Confirmed	Confirmed	hsa-mir-146a	Confirmed	Confirmed
hsa-mir-184	Confirmed	Confirmed	hsa-mir-182	Confirmed	Confirmed
hsa-mir-130a	Confirmed	Confirmed	hsa-mir-122	Confirmed	Confirmed
hsa-mir-155	Confirmed	Confirmed	hsa-mir-93	Confirmed	Confirmed
hsa-mir-20b	Confirmed	Confirmed	hsa-mir-10b	Confirmed	Confirmed
hsa-mir-29a	Confirmed	Confirmed	hsa-mir-31	Confirmed	Confirmed
hsa-mir-191	Confirmed	Confirmed	hsa-let-7g	Confirmed	Confirmed
hsa-mir-137	Confirmed	Confirmed	hsa-mir-181d	Confirmed	Confirmed

Researchers found that the second-ranked HAS-miR-96b was found to regulate apoptosis of prostate cancer cells by inhibiting the FoxO1 transcription factor, indicating that the HGCNELM subsequently validates the predictive ability of HGCNELMDA in new diseases without any known linked miRNAs. To further investigate, we set up a special case study. In this case, we examined HGCNELMDA on Lung Neoplasms, a common human cancer with many experimentally verified related miRNAs. We utilized the experimentally verified miRNA–disease associations from the HMDD v2.0 database as the initial training set. However, we removed all the associations, including lung neoplasms, from the training set this time. Hence, lung neoplasms could be regarded as a disease without any known related miRNAs. Lung tumors are devastating and fatal, causing many deaths in both males and females worldwide [41]. The survival rate of lung tumors is as low as five years, so early diagnosis is critical to save patients’ lives [43]. Therefore, lung tumors, in which miRNAs have become a promising tool in diagnosing and treating process, were selected in this case. HGCNELMDA is used to predict candidate miRNAs associated with lung tumors. The validations of the first 50 related miRNAs are listed in Table 3. Two databases confirmed 49 miRNAs, and only one miRNA was not verified. In addition, the ectopic expression of miR-494-3p in A549 lung cancer cells promoted the tumor-initiating population and enhanced the motor ability of cancer cells and the expression of stem cell-related genes, suggesting that HGCNELMDA can help the diagnosis and treatment of lung tumors. HGCNELMDA method has good accuracy in predicting prostate tumor-associated miRNA.

**Table 3:** Top 50 miRNAs associated with lung tumors

miRNA	dbDEMC	PhenomiR	miRNA	dbDEMC	PhenomiR
hsa-mir-320a	Confirmed	Confirmed	hsa-mir-28	Confirmed	Confirmed
hsa-mir-494	Confirmed	Confirmed	hsa-mir-141	Confirmed	Confirmed
hsa-mir-23b	Confirmed	Confirmed	hsa-mir-329	Confirmed	Not confirmed
hsa-mir-15a	Confirmed	Confirmed	hsa-mir-320e	Confirmed	Not confirmed
hsa-mir-107	Confirmed	Confirmed	hsa-mir-378	Confirmed	Confirmed
hsa-mir-122	Confirmed	Confirmed	hsa-mir-15b	Confirmed	Confirmed
hsa-mir-422a	Confirmed	Confirmed	hsa-mir-371	Confirmed	Confirmed
hsa-mir-377	Confirmed	Confirmed	hsa-mir-153	Confirmed	Confirmed
hsa-mir-383	Confirmed	Confirmed	hsa-mir-663	Not confirmed	Confirmed
hsa-mir-141	Confirmed	Confirmed	hsa-mir-374b	Confirmed	Confirmed
hsa-mir-342	Confirmed	Confirmed	hsa-mir-584	Confirmed	Confirmed
hsa-mir-425	Confirmed	Confirmed	hsa-mir-202	Confirmed	Confirmed
hsa-mir-377	Confirmed	Confirmed	hsa-mir-10a	Confirmed	Confirmed
hsa-mir-423	Confirmed	Confirmed	hsa-mir-16	Confirmed	Confirmed
hsa-mir-130b	Confirmed	Confirmed	hsa-mir-181d	Confirmed	Confirmed
hsa-mir-328	Confirmed	Confirmed	hsa-mir-129	Confirmed	Confirmed
hsa-mir-515	Not confirmed	Not confirmed	hsa-mir-147b	Confirmed	Confirmed
hsa-mir-320d	Confirmed	Confirmed	hsa-mir-410	Not confirmed	Confirmed
hsa-mir-323b	Confirmed	Not confirmed	hsa-mir-421	Confirmed	Confirmed
hsa-mir-92	Confirmed	Confirmed	hsa-mir-189	Confirmed	Not confirmed
hsa-mir-105	Confirmed	Confirmed	hsa-mir-17	Confirmed	Confirmed
hsa-mir-34c	Confirmed	Confirmed	hsa-mir-99a	Confirmed	Confirmed
hsa-mir-187	Confirmed	Confirmed	hsa-mir-20b	Confirmed	Confirmed
hsa-mir-149	Confirmed	Confirmed	hsa-mir-92	Confirmed	Confirmed
hsa-mir-124a	Confirmed	Confirmed	hsa-mir-302d	Confirmed	Confirmed

For the third disease case we chose pancreatic tumor as the new disease case. When the known miRNA and disease association matrix is set to zero, the column of pancreatic tumor indicates that no related miRNA is associated with it, as a new disease [43]. HGCNELMDA is used to predict candidate miRNAs associated with pancreatic tumors, and the top 50 related miRNAs are listed in Table 4. The DBDEMC and Phenomir databases validated the first 50 miRNAs. Studies have shown that increased serum miR-193b is a potential new biomarker for pancreatic neuroendocrine tumors (PNEN). The results indicate that HGCNELMDA plays an important role in predicting new diseases.

**Table 4:** Top 50 miRNAs associated with pancreatic tumors

miRNA	dbDEMC	PhenomiR	miRNA	dbDEMC	PhenomiR
hsa-mir-18a	Confirmed	Confirmed	hsa-mir-199a	Confirmed	Confirmed
hsa-let-7a	Confirmed	Confirmed	hsa-mir-210	Confirmed	Confirmed
hsa-mir-193b	Confirmed	Confirmed	hsa-mir-34c	Not confirmed	Confirmed
hsa-mir-155	Confirmed	Confirmed	hsa-mir-15a	Confirmed	Confirmed
hsa-mir-143	Confirmed	Confirmed	hsa-let-7c	Confirmed	Not confirmed
hsa-mir-19a	Confirmed	Confirmed	hsa-mir-29c	Confirmed	Confirmed
hsa-mir-29a	Confirmed	Confirmed	hsa-mir-9	Confirmed	Confirmed
hsa-mir-200c	Confirmed	Confirmed	hsa-mir-200a	Confirmed	Confirmed
hsa-mir-200b	Confirmed	Confirmed	hsa-mir-146b	Confirmed	Confirmed
hsa-mir-31	Confirmed	Confirmed	hsa-mir-182	Confirmed	Confirmed
hsa-mir-21	Confirmed	Confirmed	hsa-mir-181b	Confirmed	Confirmed
hsa-mir-155	Confirmed	Not confirmed	hsa-let-7d	Confirmed	Confirmed
hsa-mir-146a	Confirmed	Confirmed	hsa-mir-30a	Confirmed	Confirmed
hsa-mir-17	Confirmed	Confirmed	hsa-mir-142	Confirmed	Confirmed
hsa-mir-145	Confirmed	Confirmed	hsa-mir-106b	Confirmed	Confirmed
hsa-mir-20a	Confirmed	Confirmed	hsa-mir-218	Not confirmed	Confirmed
hsa-mir-34a	Confirmed	Confirmed	hsa-mir-223	Confirmed	Confirmed
hsa-mir-125b	Confirmed	Confirmed	hsa-let-7b	Confirmed	Confirmed
hsa-mir-126	Confirmed	Confirmed	hsa-let-7e	Confirmed	Confirmed
hsa-mir-221	Not confirmed	Confirmed	hsa-mir-34b	Confirmed	Confirmed
hsa-mir-92a	Confirmed	Confirmed	hsa-mir-205	Confirmed	Confirmed
hsa-mir-16	Confirmed	Confirmed	hsa-mir-7	Confirmed	Confirmed
hsa-mir-222	Confirmed	Confirmed	hsa-mir-148a	Confirmed	Confirmed
hsa-mir-181a	Confirmed	Confirmed	hsa-mir-195	Not confirmed	Confirmed
hsa-mir-29b	Confirmed	Confirmed	hsa-mir-133b	Confirmed	Confirmed

For the results of the four case studies, our method was effective when predicting unvalidated miRNA and disease interactions.

## Discussion

Compared with five classic methods based on Global LOOCV and fivefold cross-validation, the experimental results show that HGCNELMDA has better predictive performance. In addition, three case studies also support the results of our method. First, we constructed a heterogeneous network of miRNA–disease based on the proven miRNA–disease association, disease semantic similarity and miRNA functional similarity. Second, we used the restart random walk method to extract node features from similarity, aiming at reducing the data noise of extracting the original feature vectors of miRNA and disease nodes and better capturing the structural relationship between different types of nodes in the heterogeneous graph. In the miRNA–disease heterogeneous graph, to reinforce that similar nodes (miRNAs or diseases) have identical representations in the feature space, a reinforcement layer was added to the GCN hidden layer, enhancing the eigenvector aggregation of similar nodes, to preserve similar information between nodes. The attention mechanism was introduced in the reinforcement layer, more important topological neighborhood nodes were integrated, and miRNA and disease node features were extracted from the spatial topology of heterogeneous graphs to predict associations. In summary, the HGCNELMDA method makes full use of the complex structure and semantic information of the miRNA–disease heterogeneous network to achieve good predictions.

## Conclusion

This paper mainly describes the enhancement layer based heterogeneous graph convolutional network model (HGCNELMDA) to predict miRNA–disease association method. First, by restarting the random walk between the miRNA and the disease phase.

The eigenvectors of miRNA and disease nodes were obtained from the similarity network. Secondly, the heterogeneous graph of miRNA–disease was input into GCN, and a reinforcement layer was added into the hidden layer of GCN to make similar nodes have similar feature representations in the feature space. The attention mechanism was used to update the influence of important adjacent nodes in the reinforcement layer on the target node. Thirdly, the association matrix between miRNA and disease was reconstructed by bilinear encoder, and the cross-entropy loss function was used to train the model. Finally, HGCNELMDA performance was evaluated by four sets of experiments, which were left onefold cross-validation and fivefold cross-validation, compared with other methods, ablation test, parameter sensitivity test and three disease case studies. The results indicated that HGCNELMDA method had a good predictive effect in the prediction of miRNA–disease association.

## Methods

**Fig. 8:**
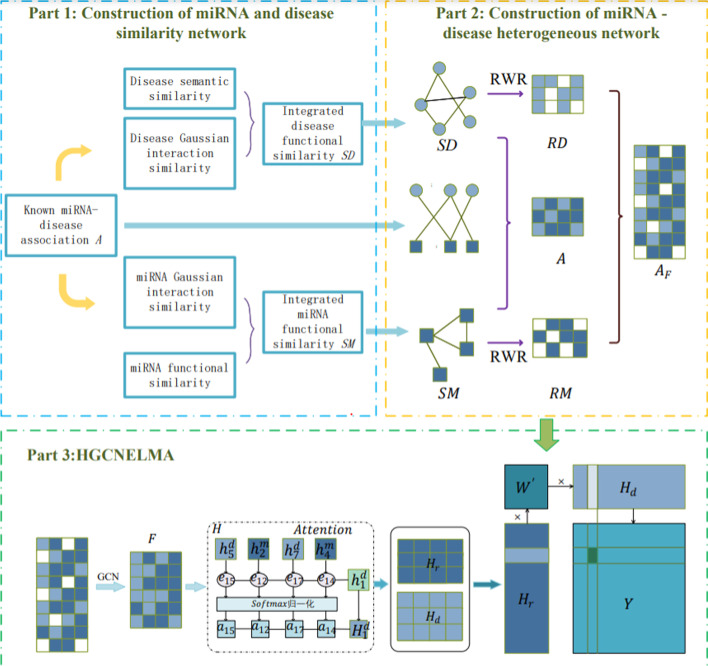
HGCNELMDA model

In order to reduce the data noise of extracting original features, make similar nodes have similar feature representation in feature space, and enhance the spatial node feature aggregation of topology map, this paper constructs a heterogeneous graph convolutional network model based on reinforcement layer to predict miRNA–disease association. The model framework is shown in Fig. 8.

(1) **Step 1:** Build miRNA–disease isomerization map according to literature [44]. Through integrated disease semantic similarity network $SD$, The known miRNA–disease association matrix is the same $A$ and an integrated miRNA functional similarity network $SM$ constructed into a miRNA–disease heterogeneous map $A_{H}$, as shown in Formula (1):

1 $${A}_{H}=\left[\begin{array}{cc}SM& A\\ {A}^{T}& SD\end{array}\right]$$

Among them, ${A}_{H}\in {\mathbb{R}}^{(m+n)\times (m+n)}$, $m$ and $n$ are respectively the number of miRNA and disease.

(2) **Step 2:** Node feature extraction based on restart random walk. In order to reduce the influence of data noise on the original features of nodes, restart the random walk is used to represent the original features of nodes.

(3) **Step 3:** Node embedding based on GCN. The information of neighbor nodes of each layer is aggregated through GCN to form an embedding of miRNA or disease node features.

(4) **Step 4:** Reinforcement layer based on attention mechanism. Since the previous GCN considered neighbor nodes equally, the text adds an attention-based reinforcement layer to the GCN hidden layer $H$.

(5) **Step 5:** Use the decoder to reconstruct the association matrix between miRNA and disease. The node feature embedding matrix is obtained by the reinforcement layer $H$, The Eigen matrix of miRNA is ${H}_{R}\in {\mathbb{R}}^{m\times h}$, The characteristic matrix of disease is ${H}_{d}\in {\mathbb{R}}^{n\times h},h$ is layer embedding dimension, Since $sigmod$ is often used as the activation function of dichotomy, It can be used to reconstruct miRNA–disease association matrix $Y$, as shown in Eq. (2):

2 $$Y=sigmod({H}_{r}{W}^{{\prime}}{H}_{d}^{T})$$

where the element in the row of the matrix $Y$ represents the correlation prediction score ${{y}_{ij}}^{{\prime}}$ between miRNA ${r}_{i}$ and disease ${d}_{j}$, ${W}^{{\prime}}\in {\mathbb{R}}^{X\times X}$ is a trainable matrix.

(6) **Step 6:** In order to make the predicted results as close as possible to the actual results, cross entropy is used as the loss function to carry out end-to-end back propagation, as shown in Formula (3):

3 $${\mathcal{L}}_{cross}=-\sum_{i,j\in y\cup {y}^{-}}({y}_{\mathit{ij}}\mathit{log}{{y}_{ij}}^{{\prime}}+\left(1-{y}_{ij}\right)\mathrm{log}\left(1-{{y}_{ij}}^{{\prime}}\right))$$

Among them, $y$ represents an associated miRNA–disease positive sample, ${y}^{-}$ represents a negative sample with an unknown relationship.

### Feature extraction based on random walk with restart

The M2GMDA and CEMDA methods assign each row or column in the $SM$ (or $SD$) similarity matrix to represent an eigenvector of amiRNA (or disease). Literature [45] believes that the limitation of similarity calculation method may lead to some data noise in the direct extraction of original node features. Therefore, in order to optimize the original feature vectors of miRNA and disease nodes and better capture the structural relationship between different types of nodes in heterogeneous graphs, the text reference uses a method based on R (random walk with restart, RWR) to extract node features from similarity. Restarting the random walk starts from a node, and each step can randomly select adjacent nodes or return to the starting node. Assume that there are $n$ nodes, Right at the start node $e$, then the probability of appearing at any node $i$ in the next move is $r$, as shown in Formula (4):

4 $${r}^{0}\left[i\right]=W\left[i\right]\cdot e$$

Here, $W\left[i\right]$ represents the *i* row of the transition probability matrix $W$, that is, the transition probability from all nodes to node $i$, in the next move, the probability of the node $i$ is shown in formula (5):

5 $${r}^{1}\left[i\right]=W\left[i\right]\cdot {r}^{0}$$

After considering restarting, after $k$ iterations, it still returns to node $i$ with probability $c$. After the $k+1$ iteration is stable, ${r}_{i}$ is the probability score of reaching node $i$, which is the similarity feature vector of node $i$, as shown in formula (6):

6 $${r}_{i}^{k+1}=cW{r}_{i}^{k}+\left(1-c\right){e}_{i}$$

Here, $c\in \left(0, 1\right)$ represents the restart probability, $W\left[i,j\right]\in {\mathbb{R}}^{n\times n}$ represents the probability from $i$ to $j$, and ${e}_{i}\in {\mathbb{R}}^{n\times 1}$ is the $i$-th node Initial probability vector. If $i$ is equal to $j$, then ${e}_{ij}$ is 1, otherwise it is 0. This paper replaces $W$ with $SM$ or $SD$ respectively, and obtains the probability distribution matrix of the node (miRNA or disease) based on the restart random walk, and normalizes the feature matrix as the miRNA feature matrix $RM\in {\mathbb{R}}^{m\times m}$ and the characteristic matrix of the disease $RD\in {\mathbb{R}}^{n\times n}$. By restarting the random walk, the similarity between two points can be obtained, and the global structure of the graph can be better captured. According to $RM$ and $RD$, the characteristic matrix of miRNA–disease ${A}_{F}{\in {\mathbb{R}}}^{(m+n)\times (m+n)}$ is obtained, as shown in formula (7):

7 $${A}_{F}=\left[\begin{array}{cc}0& RM\\ RD& 0\end{array}\right]$$

### GCN-based node cutting

Graph convolution aggregates node information according to edge information and represents new node features. The two feature extraction methods of graphs are spatial domain and Spectral domain. According to the explanation in Literature [44], the spatial method means that the neighbor nodes connected with the vertex are directly used to extract features. But the spectral method hopes to realize the convolution operation on the graph with the help of the graph theory, and studies the properties of the graph with the eigenvalues and eigenvectors of the Laplace matrix of the graph. Laplacian matrices are symmetric matrices, and GCN can perform feature decomposition. Common Laplacian matrix is symmetric normalized Laplacian, each node is the purpose of the normalized Laplacian matrix by foreign transfer the same amount of information, the more edge nodes exist, the less the amount of information transmitted each edge. The definition of the symmetric normalization Laplace matrix is shown in Eq. (8):

8 $$\widehat{L}={D}^{-\frac{1}{2}}\cdot L\cdot {D}^{-\frac{1}{2}}$$

Here, $D$ represents the degree matrix of the vertex, also called the diagonal matrix, and the definition of the elements of the $L$ matrix is shown in formula (9):

9 $${L}_{ij}=\left\{\begin{array}{ll}1&\quad i=j\, and\, diag({v}_{i})\ne 0\\ -\frac{1}{\sqrt{diag\left({v}_{i}\right)diag\left({v}_{j}\right)}} &\quad i\ne j \,and\, {v}_{i}\, is\, adjacent \,to\, {v}_{j} \\ 0& \quad otherwise\end{array}\right.$$

According to the heterogeneous map ${A}_{H}$ of miRNA–disease, the normalized Laplacian matrix is constructed as shown in formula (10):

10 $${\widehat{A}}_{H}{=D}^{-\frac{1}{2}}{ A}_{H}{ D}^{-\frac{1}{2}}$$

Literature [45] indicates that Laplace matrix and Fourier transform are the two theoretical foundations of GCN. The Fourier transform of the graph expresses the arbitrary vector $f$ defined on the graph as a linear combination of the eigenvectors of the Laplacian matrix, as shown in formula (11):

11 $$f=\widehat{f}\left(1\right){u}_{1}+ \widehat{f}\left(2\right){u}_{2}+\dots \widehat{f}\left(n\right){u}_{n}$$

$({u}_{1},{u}_{2},\cdot \cdot \cdot {u}_{n})$ is a set of orthogonal bases formed by $n$ linearly independent vectors. The relationship between Fourier transform and Laplace matrix: The eigenvector of Laplace matrix is the base of Fourier transform, Get the graph convolution network, as shown in formula (12):

12 $$f(X,A)=ReLU(\widehat{A}XW)$$

Here, $X$ represents the feature matrix of the node, $\widehat{A}$ represents the normalized adjacency matrix, and $W$ is the weight matrix from the input layer to the hidden layer, which is equivalent to using a fully connected network to combine the feature connections.

According to the miRNA–disease heterogeneous map ${A}_{H}$ and the miRNA–disease feature matrix ${A}_{F}$, the initial embedding of miRNA and disease nodes is formed through GCN. Make GCN directly connect and gather the information of neighbor nodes on each layer through the graph, as the input of the next layer, as shown in formula (13):

13 $$F=f({A}_{F},{A}_{H})=ReLU({ \widehat{A}}_{H}{A}_{F}{W}^{(0)})$$

Here, ${W}^{(0)}{\in {\mathbb{R}}}^{(m+n)\times h}$, $h$ embeds dimensions for layers.

### Reinforcement layer based on attention mechanism

In order to make similar miRNA (or disease) nodes similar in the feature space, this paper added an attentional strengthening layer $H$ into the GCN hiding layer. The initial reinforcement layer $H$ was defined as $F$, and an attention mechanism was introduced to consider all neighbor nodes. The attention mechanism is used to measure the influence of the feature vector $H$ of adjacent nodes in the reinforcement layer on the feature vector $H$ of nodes. ${a}_{ij}$ represents the attention coefficient between nodes, as shown in Formula (14), (15) and (16):

14 $${e}_{ij}=ReLU\left(W{h}_{i},W{h}_{j}\right)$$

15 $${a}_{ij}=\frac{\mathrm{exp}({e}_{ij})}{{\sum }_{j\in {\mathcal{N}}_{i}}\mathrm{exp}({e}_{ix})}$$

16 $${H}_{i}={\sum }_{j\in {\mathcal{N}}_{i}}{a}_{ij}{h}_{i}$$

where ${\mathcal{N}}_{i}$ is the set of neighborhood nodes of node $i$. $ReLU$ is the activation function and $W{\in {\mathbb{R}}}^{(m+n)\times X}$ is a trainable matrix.

Next, define the $Loss$ function ${\mathcal{L}}_{H}$ of the reinforcement layer. In order to make the feature vector of node ${H}_{i}$ on the reinforcement layer $H$ focus on the feature vector ${H}_{j}$ of important similar neighbor nodes, so that the feature vector of node $i$ can be better iterated and updated, $Loss$ function is defined as follows, as shown in Eq. (17)

17 $$Loss\left({H}_{i}\right)=\sum_{i=1}^{m+n}{\sum }_{j\in {N}_{i}}{a}_{ij}{|{H}_{i}-{H}_{j}|}^{2}$$

Among them, *m* and *n* represent the number of miRNAs and diseases.

## Data Availability

The datasets that support the findings of this study are available in https://github.com/liubailong/HGCNELMDA.

## Abbreviations

HGCNELMDA: Heterogeneous graph convolutional network model with enhanced layer to predict miRNA–disease associations
GCN: Graph convolutional network
RWR: Random walk with restart
LOOCV: Global leave-one-out cross validation
miRNAs: Micro ribonucleic acids
AUC: Area under the curve
